# Novel flax orbitide derived from genetic deletion

**DOI:** 10.1186/s12870-018-1303-8

**Published:** 2018-05-21

**Authors:** Peta-Gaye Gillian Burnett, Lester Warren Young, Clara Marisa Olivia, Pramodkumar Dinkar Jadhav, Denis Paskal Okinyo-Owiti, Martin John Tarsisius Reaney

**Affiliations:** 1 Department of Plant Sciences, College of Agriculture and Bioresources, University of Saskatchewan, Saskatoon, SK S7N 5A8 Canada; 2 Department of Food and Bioproduct Sciences, College of Agriculture and Bioresources, University of Saskatchewan, Saskatoon, SK S7N 5A8 Canada; 3 Guangdong Saskatchewan Oilseed Joint Laboratory, Department of Food Science and Engineering, Jinan University, Guangzhou, 510632 Guangdong China

**Keywords:** *Linum usitatissimum* L., Orbitides, Cyclolinopeptides, Plant genetic resources, Mass spectrometry, Sequencing

## Abstract

**Background:**

Flaxseed orbitides are homodetic plant cyclic peptides arising from ribosomal synthesis and post-translation modification (N to C cyclization), and lacking cysteine double bonds (Nat Prod Rep 30:108-160, 2013). Screening for orbitide composition was conducted on the flax core collection (FCC) grown at both Saskatoon, Saskatchewan and Morden, Manitoba over three growing seasons (2009-2011). Two flax (*Linum usitatissimum* L.) accessions ‘Hollandia’ (CN 98056) and ‘Z 11637’ (CN 98150) produce neither [1−9-N*α*C]-linusorb B2 (**3**) nor [1−9-N*α*C]-linusorb B3 (**1**). Mass spectrometry was used to identify novel compounds and elucidate their structure. NMR spectroscopy was used to corroborate structural information.

**Results:**

Experimental findings indicated that these accessions produce a novel orbitide, identified in three oxidation states having quasimolecular ion peaks at *m/z* 1072.6 (**18**), 1088.6 (**19**), and 1104.6 (**20**) [M + H]^+^ corresponding to molecular formulae C_57_H_86_N_9_O_9_S, C_57_H_86_N_9_O_10_S, and C_57_H_86_N_9_O_11_S, respectively. The structure of **19** was confirmed unequivocally as [1−9-N*α*C]-*O*LIPPFFLI. PCR amplification and sequencing of the gene coding for **18**, using primers developed for **3** and **1**, identified the putative linear precursor protein of **18** as being comprised of the first three amino acid residues of **3** (MLI), four conserved amino acid residues of **3** and/or **1** (PPFF), and the last two residues of **1** (LI).

**Conclusion:**

Comparison of gene sequencing data revealed that a 117 base pair deletion had occurred that resulted in truncation of both **3** and **1** to produce a sequence encoding for the novel orbitide precursor of **18**. This observation suggests that repeat units of flax orbitide genes are conserved and suggests a novel mechanism for evolution of orbitide gene diversity. Orbitides **19** and **20** contain MetO and MetO_2_, respectively, and are not directly encoded, but are products of post-translation modification of Met present in **18** ([1−9-N*α*C]-MLIPPFFLI).

**Electronic supplementary material:**

The online version of this article (10.1186/s12870-018-1303-8) contains supplementary material, which is available to authorized users.

## Background

**Fig. 1:**
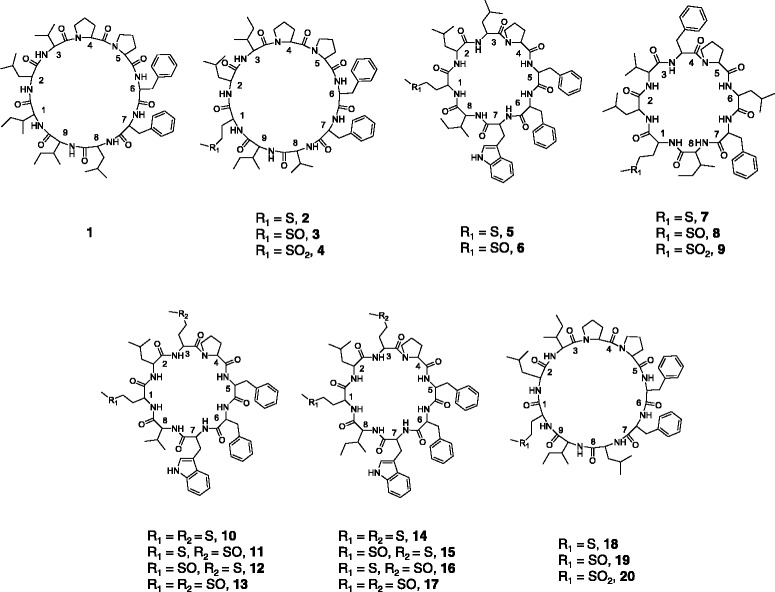
Structures of known and novel flaxseed orbitides observed in this study

Orbitides can be found in plant families and genera other than Linaceae and *Linum*, including Annonaceae (*Panax*), Caryophyllaceae (*Saponaria)*, Euphorbiaceae (*Jatropha*), and Rutaceae (*Citrus*) [1–3]. Although their biological function is unknown, they are thought to serve an important role due to their abundance and conserved nature. Orbitides, like other macrocylic peptides, are resistant to protease digestion due to covalently closed ends which may be critical for biological activity [4]. Potential biological activities of flax orbitides and their analogs have been investigated over the past two decades [5–16] and the results summarized in a review [17]. To date, eleven parent flaxseed orbitides (**1**, **2**, **5**, **7**, **10**, **14**, **21**, **23**-**25** and **27)** have been discovered and the corresponding DNA sequences encoding precursor proteins have been identified [18–20] from the flax genome database [21] (Fig. 1, Additional file 1: Table S1). The remaining flax orbitides reported in the literature (**3**, **4**, **6**, **8**, **9**, **11**-**13**, **15**-**17**, **22**, **26** and **28**) result from methionine post-translational oxidation.

All linear proteins containing orbitide domains are encoded by genes. These linear precursor proteins share structural similarities. In *L. usitatissimum*, orbitide domains are flanked predominantly by “DD”, “DG” or “SD” residues at the amino termini, and “FGK” or “VGK” residues at the carboxy termini. Additionally, some of these orbitide domains are present as tandem repeats in the proprotein carboxy terminal. The orbitide precursor protein of **5**, **10**, and **14** has been identified in *L. usitatissimum* chromosome Lu14 (National Center for Biotechnology Information (NCBI) GenBank™ CP027624.1 residues 17099153 to 17099545 [21]; as these orbitides are coded by the same gene it is hereafter named *LINUSORB A*, following our proposed systematic nomenclature [22]) for example. The function of the first exon of this gene is unknown, while the second exon encodes a linear precursor protein with five orbitide domains arranged in repeats in the order **14**, **14**, **5**, **14**, **10**. The conserved nature of sequences flanking the orbitide coding region and occurrence of repeat units has allowed the identification of several other related putative orbitides in *Citrus* and *Saponaria* [2]. Additional gene sequences that appear to code for flax orbitides have been identified based on their conserved tandem repeat structure. However, detection of the actual orbitides remains to be established. It is not known in which tissue the gene may be expressed, or if the apparent genes are expressed at all.

## Results

Recently, orbitide compositions of the flax core collection (FCC) harvested in 2009 were determined using high-performance liquid chromatography with reversed-phase monolithic HPLC columns and diode array detection (HPLC-DAD) [23]. The FCC is a subset of the Canadian flax world collection curated by Plant Gene Resources of Canada (PGRC) comprising 381 accessions [24]. The FCC was assembled to represent the phenotypic diversity and genetic variability of approximately 3500 flax accessions from around the world held in the Canadian collection. The FCC currently includes 407 accessions [25]. An additional ten cultivars were included in the study of orbitides in the FCC, namely ‘CDC Sorrel’, ‘CDC Mons’, ‘Lightning’, ‘Prairie Blue’, ‘Prairie Grande’, ‘Prairie Thunder’ and ‘Shape’ along with the checks ‘CDC Bethune’, ‘Hanley’, and ‘Macbeth’. Screening was limited to orbitides **1**, **3**, and **8** (encoded by the gene contained within *L. usitatissimum* chromosome Lu11; National Center for Biotechnology Information (NCBI) GenBank™ Sequence ID: CP027621.1 residues 2516493 to 2517107 [21]; Hereafter, *LINUSORB B* following our proposed systematic nomenclature [22]) and orbitides **6**, **13**, and **17** (encoded by *LINUSORB A*). These orbitides were chosen because of their high expression and known chromatographic separation methods. ‘Hollandia’ (CN 98056) and ‘Z 11637’ (CN 98150), both originated from The Netherlands, and were identified as accessions expressing the lowest total orbitide content and the lowest apparent concentration ratio of orbitides [**1**, **3**, **8**] to [**6**, **13**, **17**]. Closer evaluation revealed that the differences were attributed to a lack of **1** and **3**, while the apparent concentration of **8** was comparable to the mean of the core collection. Expression of **6**, **13** and **17** in these accessions was not significantly different from the rest of the core collection.

**Fig. 2:**
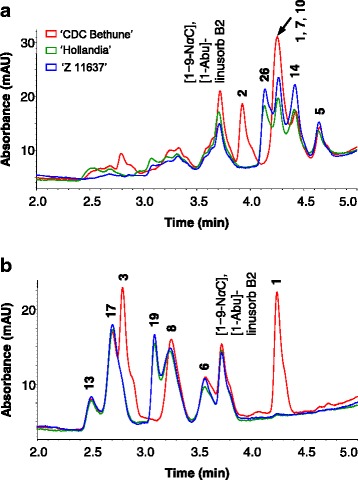
HPLC-DAD chromatograms of flaxseed (*L. usitatissimum*) ‘CDC Bethune’, ‘Hollandia’, and ‘Z 11637’ orbitide extracts. **a** unoxidized and **b** oxidized

Orbitide compositions of oxidized extracts from accessions ‘Hollandia’ and ‘Z 11637’ were compared in detail with orbitides of ‘CDC Bethune’. The HPLC-DAD chromatograms displayed elution peaks at approximately 2.5, 2.7, 2.8, 3.3, 3.6, and 4.3 min corresponding to **13**, **17**, **3**, **8**, **6**, and **1**, respectively (Fig. 2a). Orbitides **1** and **3** were either not detected or were detected at low concentrations in ‘Hollandia’ and ‘Z 11637’, while expression of **8** in these accessions was comparable to that of ‘CDC Bethune’. Hence, orbitide concentrations of **1**:**3**:**8** observed in ‘Hollandia’ and ‘Z 11637’ deviated from the 1:1:1 ratio expressed in ‘CDC Bethune’. These findings were consistent regardless of harvest location (Saskatoon, SK or Morden, MB) and year (2009, 2010, 2011) and corroborate observations made by Burnett et al. [23] (Table 1). Low level detection of **1** and **3** in ‘Hollandia’ and ‘Z 11637’ resulted from flaxseed samples that were contaminated with one or more other accessions within the FCC. Such contamination is not uncommon in field plot studies. In 2009, mean total orbitide contents of ‘Hollandia’, ‘Z 11637’ and ‘CDC Bethune’ were 139.2, 106.9 (Table 1) and 196.9 mAU [23]. Similar total orbitide concentrations in ‘Hollandia’ and ‘Z 11637’ were observed in 2010 and 2011 with mean 2010 values being lower probably due to poor seed quality from the Saskatoon plots that year. Plant maturity at harvest was atypical because of a short growing season (from delayed seeding) with excess moisture. Overall, the sum of apparent orbitide concentrations of **1**, **3**, and **8** (encoded by *LINUSORB B*), hereafter refered to as [**1**,**3**,**8**], were lower than those of **6**, **13**, and **17** (encoded by *LINUSORB A*), hereafter refered to as [**6**,**13**,**17**]. The mean apparent concentration ratio of [**1**,**3**,**8**] to [**6**,**13**,**17**] observed in accessions ‘Hollandia’ and ‘Z 11637’ over three growing seasons was 0.7 ± 0.2, while that observed in ‘CDC Bethune’ in 2009 was 1.9 ± 0.1 [23]. These ratios were consistent regardless of harvest location and reflected the apparent low total orbitide expression of these accessions.

**Table 1:** Average orbitide distribution in oxidized aqueous methanolic extracts of ‘Hollandia’ and ‘Z 11637’ flaxseed accessions

Orbitide (mAU)	‘Hollandia’			‘Z 11637’		
	2009	2010	2011		2010	2011
1	2.0 ± 2.4	7.9 ± 9.2	10.7 ± 4.6	0.0 ± 0.0	0.9 ± 1.2	2.2 ± 2.5
3	1.7 ± 1.9	8.1 ± 9.5	10.1 ± 4.8	0.0 ± 0.0	1.3 ± 1.5	1.4 ± 1.6
6	19.1 ± 4.4	14.1 ± 0.6	17.0 ± 1.8	15.5 ± 3.2	13.7 ± 5.0	17.7 ± 1.9
8	48.2 ± 22.2	38.1 ± 1.3	39.8 ± 2.3	36.2 ± 5.8	34.3 ± 7.2	35.3 ± 2.8
13	16.9 ± 3.1	13.5 ± 1.2	15.2 ± 0.9	13.5 ± 2.2	9.8 ± 4.3	13.9 ± 1.0
17	51.2 ± 10.6	38.5 ± 2.8	43.8 ± 3.2	41.7 ± 6.3	31.9 ± 13.1	44.4 ± 2.3
19	38.6 ± 7.3	29.8 ± 2.5	28.3 ± 6.9	35.4 ± 5.3	29.4 ± 6.2	31.9 ± 2.7
Total orbitide	139.2 ± 33.4	121.4 ± 21.8	135.0 ± 6.5	106.9 ± 17.4	91.9 ± 26.9	114.9 ± 5.8
[1,3,8]/[6,13,17]	0.6 ± 0.2	0.8 ± 0.3	0.8 ± 0.1	0.5 ± 0.0	0.7 ± 0.2	0.5 ± 0.0

Data are presented as mean values (peak area in mAU) from replicate analyses from named flaxseed accessions grown in Saskatoon, SK and Morden, MB plots; *n* = 4

An unidentified compound, at retention time 3.1 min, (**18**) was detected in HPLC-DAD chromatograms of oxidized orbitide extracts from ‘Hollandia’ and ‘Z 11637’, suggesting the presence of a novel compound (Fig. 2a). Orbitide extracts from these two accessions were analyzed without addition of hydrogen peroxide to determine whether the new peak corresponded to an oxidation product or to a compound unaffected by oxidation. The HPLC-DAD analyses of unoxidized extracts of ‘CDC Bethune’ showed the presence of **2**, **14** and **5** eluting at 3.9, 4.4 and 4.7 min, respectively, along with the co-elution of **1**, **7** and **10** at 4.3 min (confirmed by HPLC-ESI-MS and HPLC-ESI-MS/MS analyses) (Fig. 2b). Stefanowicz [26], Okinyo-Owiti et al. [20], and Burnett et al. [23] have reported similar observations for orbitide content of fresh flaxseed extracts. Unoxidized flaxseed extracts from ‘Hollandia’ and ‘Z 11637’ contained known orbitides **7**, **10**, **14**, and **15** as found in ‘CDC Bethune’. However, in contrast to “CDC Bethune’, (i) no peak was observed for **2**; (ii) the absence of **1** was confirmed by mass spectrometry; and (iii) an additional peak at retention time 4.2 min (**18**) was observed in extracts from ‘Hollandia’ and ‘Z 11637′ (Fig. 2b). The lack of **1** and **3** in oxidized extracts was supported by the absence of **1** and **2** in unoxidized extracts as **1** is unaffected by oxidation, while **3** is derived from post-translational oxidation of the methionine (Met) residue in **2** to methionine *S*-oxide (MetO). HPLC-DAD analyses of ‘Hollandia’ and ‘Z 11637′ grown at two locations over three years confirmed the consistent presence of the new peak at an apparent concentration of 32.1 ± 5.0 mAU. Signals in both oxidized and unoxidized extracts are consistent with a novel orbitide (**18)** and this orbitide is prone to oxidation.

**Fig. 3:**
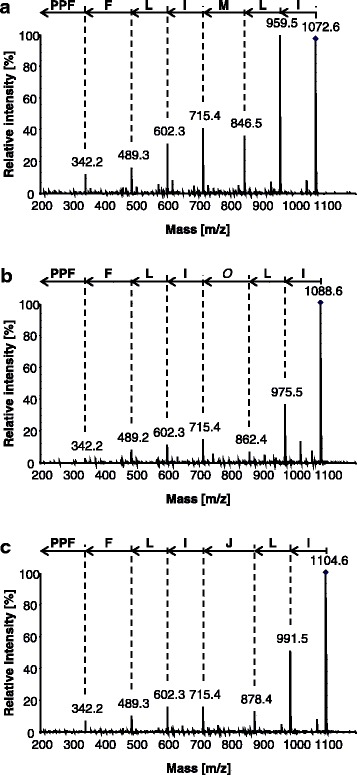
Product ion spectra of ions derived from 18, 19, and 20. **a** [1−9-N*α*C]-MLIPPFFLI (18, *m/z* 1072.6246) at *t*_R_ 7.4 min, **b** [1−9-N*α*C]-*O*LIPPFFLI (19, *m/z* 1088.6211) at *t*_R_ 3.7 min, and **c** [1−9-N*α*C]-JLIPPFFLI (20, *m/z* 1104.6150) at *t*_R_ 5.7 min

Oxidized and unoxidized extracts of ‘Hollandia’, ‘Z 11637’ and ‘CDC Bethune’ were subjected to extensive HPLC-ESI-MS and HPLC-ESI-MS/MS analyses. The unidentified peak (**18**) occurring in unoxidized extracts possessed a quasimolecular ion at *m/z* 1072.6246 ([M + H]^+^, *t*_R_ = 7.4 min). Tandem mass spectrometry displayed ring opening at the amide nitrogen of proline followed by sequential loss of neutral single amino acid residues typical of b fragmentation. The proposed mass fragmentation pattern of **18** is: Leu/Ile-Leu/Ile-Met-Leu/Ile-Leu/Ile-Phe to *m/z* 342.18 ([M + H]^+^ which is attributed to the tripeptide fragment [Phe-Pro-Pro] (Fig. 3a). The HPLC-ESI-MS spectra of oxidized extracts from the anomalous accessions showed novel orbitide **19** (*m/z* 1088.6211 ([M + H]^+^, *t*_R_ = 3.7 min), in addition to low apparent concentration of **20** (*m/z* 1104.6150 ([M + H]^+^, *t*_R_ = 5.7 min). Both **19** and **20** showed similar fragmentation patterns to that of **18**, with the exception of the third, methionine-containing, residue (Figs. 3b and c). The third cleavage residue observed in these spectra corresponded to a loss of 147.04 Da and 163.03 Da in **19** and **20**, respectively. The difference (15.9949 Da) between **18** and **19**, and between **19** and **20**, suggests that **19** and **20** were derived from oxidative modification of Met present in **18** to MetO and MetO_2_, respectively. Oxidation of the Met residue to MetO and subsequently to MetO_2_ arose from addition of H_2_O_2_ to the extracts. Based on these assumptions, the molecular weights and tandem mass spectra, the proposed mass fragmentation pattern of **19** is Leu/Ile-Leu/Ile-MetO-Leu/Ile-Leu/Ile-Phe-[Phe-Pro-Pro], while that of **20** is Leu/Ile-Leu/Ile-MetO_2_-Leu/Ile-Leu/Ile-Phe-[Phe-Pro-Pro].

**Fig. 4:**
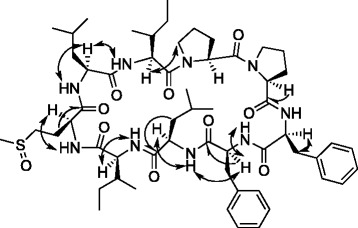
Structure of 19 ([1−9-N*α*C]-*O*LIPPFFLI). Double arrows show selected NOESY correlations, while half arrows show selected HMBC correlations

Orbitide **19** was isolated in sufficient quantities for further characterization via nuclear magnetic resonance (NMR). NMR spectra were conducted in deuterated chloroform and recorded at 308 K (Additional file 2: Figure S1, Additional file 3: Figure S2, Additional file 4: Figure S3, Additional file 5: Figure S4, Additional file 6: Figure S5, Additional file 7: Figure S6, Additional file 8: Figure S7 and Additional file 9: Figure S8). Sequential assignment of *α*, *β*, *γ* and *δ* protons were performed using ^1^H-^1^H correlation spectroscopy (COSY), total correlation spectroscopy (TOCSY) and Nuclear Overhauser effect spectroscopy (NOESY) experiments. In addition, heteronuclear multiple quantum correlation (HMQC) was performed to assign carbon atoms attached to protons. Resonances arising from protons attached to heteroatoms were assigned using ^1^H NMR data and their coupling to amide protons and carbonyl carbons employed NOE and heteronuclear multiple-bond correlation (HMBC) spectroscopy, all of which collectively confirmed the orbitide amino acid sequence. The NMR spectral data of **19** (Fig. 4, Table 2) showed remarkable similarities to that of **3** which is not surprising as the calculated molecular weight of **19** (*m/z* 1088.6213 ([M + H]^+^, C_57_H_86_N_9_O_10_S) is 14.0157 Da higher than that of **3** (*m/z* 1074.6056 ([M + H]^+^, C_56_H_84_N_9_O_10_S, [1−9-N*α*C]-*O*LIPPFFVI) (Additional file 1: Table S1), thereby suggesting the presence of an additional methylene moiety [9]. ^1^H NMR data of **19** displayed most resonances also observed in that of **3**. However, the ^1^H NMR spectrum of **19**, unlike **3**, displayed two sharp singlets at *δ* 2.54 and 2.56 ppm and carbon signals at *δ* 38.17 and 38.73 ppm that correspond to methyl of a MetO moiety. Note that **3** in deuterated dimethyl sulfoxide exhibited only one MetO singlet at *δ* 2.45 ppm and a carbon signal at *δ* 37.6 ppm [9]. These observations suggest that **19** existed in two stable conformers under the NMR experimental conditions employed and that the MetO exists in R or S diastereomeric forms. The ^1^H NMR revealed seven amide proton signals (*δ* 6.74, 6.95, 7.45, 7.48, 7.65 (2), and 7.76 ppm) (Table 2). The ^13^C NMR spectrum showed two sets of signals for each carbon atom corresponding to two conformers; signals from the major conformer are shown in Table 2. Nine amide carbonyl signals (*δ* 170.69, 170.77, 171.14, 171.40, 171.50, 172.42, 172.74, 172.98, and 173.36 ppm) were observed in ^13^C NMR indicating that **19** is a nonapeptide. NMR analyses (Table 2) showed the presence of Leu in **19** instead of Val as detected in **3** [9]. The NMR spectroscopic data clearly distinguishes between Leu and Ile residues and further corroborated mass spectral analyses thus confirming the structure of **19** as: Ile-Leu-MetO-Ile-Leu-Phe-Phe-Pro-Pro.

**Table 2:** ^1^H and ^13^C NMR assignments for [1−9-N*α*C]-*O*LIPPFFLI (**19**)

Assignment		δ_H_	δ_C_	Assignment		δ_H_	δ_C_
MetO^1^				Phe^6^			
	*α*	4.78 (1H, m)	52.11		*α*	4.78 (1H, m)	54.78
	*β*	2.25 (2H, m)	29.93		*β*	2.95 (2H, m)	36.01
	*γ*	3.02 (2H, m)	50.09		*γ*		136.61
	*ε*_Me_	2.54 (3H, s)	38.17		*δ*		129.71
	NH	7.76 (1H, s)			*ε*		129.02
	C=O		173.36		*ζ*		126.84
Leu^2^					NH	7.45 (1H, m)	
	*α*	4.51 (1H, m)	55.82		C=O		172.74
	*β*	2.25 (1H, m)	40.38	Phe^7^			
		1.97 (1H, m)			*α*	4.87 (1H, m)	54.12
	*γ*	1.62 (1H, m)	28.55		*β*	3.08 (2H, m)	34.59
	*δ*_Me_	0.93 (3H, m)	23.21		*γ*		136.95
		0.88 (3H, m)	21.56		*δ*		129.97
	NH	6.95 (1H, m)			*ε*		129.05
	C=O		170.77		*ζ*		127.24
Ile^3^					NH	7.65 (1H, m)	
	*α*	4.58 (1H, m)	56.15		C=O		171.14
	*β*	1.94 (1H, m)	36.89	Leu^8^			
	*γ*	1.69 (2H, m)	24.34		*α*	4.08 (1H, m)	54.98
	*γ*_Me_	1.07 (3H, m)	15.65		*β*	2.15 (1H, m)	38.73
	*δ*_Me_	0.93 (3H, m)	11.31			1.91 (1H, m)	
	NH	8.06 (1H, br.s)			*γ*	1.62 (1H, m)	25.03
	C=O		171.40		*δ*_Me_	0.97 (3H, m)	23.34
Pro^4^						0.94 (3H, m)	21.74
	*α*	3.98 (1H, m)	59.03		NH	7.65 (1H, br.s)	
	*β*	2.28 (1H, m)	28.78		C=O		171.50
		2.06 (1H, m)					
	*γ*	1.94 (1H, m)	24.68				
		1.69 (1H, m)		Ile^9^			
	*δ*	3.94 (1H, m)	48.06		*α*	4.34 (1H, m)	58.71
		3.69 (1H, m)			*β*	1.99 (1H, m)	36.73
	C=O		170.69		*γ*	1.51 (2H, m)	25.37
Pro^5^					*γ*_Me_	0.94 (3H, m)	16.02
	*α*	4.13 (1H, m)	61.28		*δ*_Me_	0.88 (3H, m)	11.78
	*β*	1.94 (2H, m)	32.11		NH	6.74 (1H, m)	
	*γ*	1.51 (1H, m)	21.99		C=O		172.42
		1.14 (1H, m)					
	*δ*	3.30 (2H, m)	47.39				
	C=O		172.98				

Orbitides, by definition, are ribosomally synthesized and post-translationally modified N-to-C linked peptides. Consequently, compounds **18**, **19**, and **20** cannot be unequivocally classified as orbitides until their corresponding biosynthetic precursor is identified. Based on the mass fragmentation pattern of **19** and its homology with **3**, the proposed sequence of the mature genetically encoded **18** is [1−9-N*α*C]-MLIPPFFLI. Based on the recently proposed systematic nomenclature of flax orbitides [17, 22], novel orbitides **18** ([1−9-N*α*C]-MLIPPFFLI), **19** ([1−9-N*α*C]-*O*LIPPFFLI), and **20** ([1−9-N*α*C]-JLIPPFFLI) are designated [1−9-N*α*C]-linosorb F1, [1−9-N*α*C]-[1-MetO]-linosorb F1, [1−9-N*α*C]-[1-MetO_2_]-linosorb F1, respectively. Although an assembly of the entire *L. usitatissimum* genome has been published [21], the genomic data is constructed only from the cultivar ‘CDC Bethune’. Nevertheless, we conducted TBLASTN [27, 28] searches using default algorithm parameters with a putative precursor for **18,** MLIPPFFLI, as the initial query in addition to all possible linear transcripts for **18**. The searches returned no hits which is not surprising given that the observed changes in orbitide production in ‘Hollandia’ and ‘Z 11637’ indicate that these accessions may contain different sequences and/or may differ in their transcription, translation, and/or post-translational modification of *LINUSORB B* compared to ‘CDC Bethune’.

*LINUSORB B* encodes a gene that contains one copy of each of the linear precursor orbitides of **1**, **3** and **8**. These orbitides are expressed equally in ‘CDC Bethune’, as in all other accessions within the FCC with the exception of ‘Hollandia’ and ‘Z 11637’ [23]. We studied the orbitide encoding region of *LINUSORB B* to determine if an alteration in this gene was responsible for the unusual apparent concentration ratio of orbitides and low total orbitide content observed in ‘Hollandia’ and ‘Z 11637’. Sequencing of the orbitide encoding region of this gene in ‘Hollandia’ and ‘Z 11637’ revealed a 117 bp deletion (Additional file 10). The transcribed gene encodes an orbitide with amino acid sequence MLIPPFFLI and corresponds to the deduced structure of **18**. As the size of the orbitide peptides repeat unit is 117 bp, the deletion maintains the reading frame of the gene and results in the synthesis of two orbitides (**18** and **7**) instead of three (**1**, **2** and **7**).

## Discussion

**Fig. 5:**
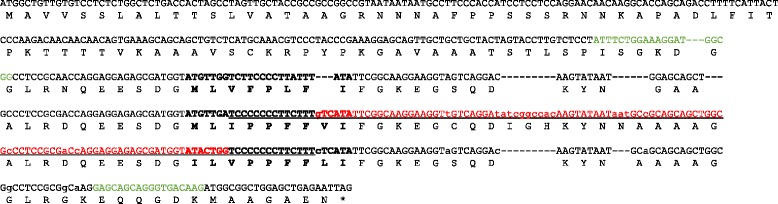
Open reading frame of *LINUSORB B*, which encodes Orbitides **1**, **2**, and **7**. Sequence arranged so that repeats within gene are aligned (nucleotide lines 3-5). Dashes are inserted to allow alignment of repeats. Bolded: Orbitides **7**, **2** and **1** from top to bottom, respectively. Green text: location of PCR primers. Red text: Deleted sequence in ‘Hollandia’/‘Z11637’. Underlined: identical sequence between Orbitides **2** and **1** indicating the region for the start and end of the 117 bp deletion. Lowercase: Differences between sequence following Orbitide **2** and sequence following Orbitide **1** up to the primer binding site

The deletion precludes expression of intact **1** and **2**, but results in the joining of the 5′ fragments of **2** and the 3′ portion of **1** to form the novel orbitide **18**. Orbitides **1** and **2** have fourteen bp in common (TCCCCCCCTTCTTT); the beginning and end of the deletion occurs within this shared sequence for **2** and **1**, respectively (Fig. 5). It is not possible to determine the exact nucleotide at which the deletion starts and ends. However, the peptide sequences of Orbitides **1, 2** and **18** are conserved at this location for the ‘Hollandia’, ‘Z11637’ and other genotypes. This means that the lesion resulting in Orbitide **18** occurred at the same nucleotide in this region for Orbitides **1** and **2** (Fig. 5).

Evidence that the 117 bp deletion occurred between the sequences for Orbitides **2** and **1** is that the sequence of the PCR fragment from ‘Hollandia’ and ‘Z11637’ situated 5′ of Orbtide **2** and 3′ of Orbitide **1** are identical to that of the reference gene sequence from CDC Bethune and from PCR fragments amplified from 16 other genotypes (data not shown). Furthermore, sequence differences unique to the deleted fragment (7 nucleotide differences and a 3 bp and a 9 bp insertion) were not observed in the sequence of the PCR product from ‘Hollandia’ and ‘Z11637’. Taken together, the simplest explaination for the observed DNA and orbitide sequence in ‘Hollandia’ and ‘Z11637’ is a 117 bp deletion starting in the common 14 bp region of Orbitide **2** and ending at the same nucleotide in Orbitide **1**. Alternative explanations are possible, but are not as parsimonious as the one suggested here as they need to explain the conserved sequence identity in the rest of the PCR fragment and the absence of the unique sequence differences located between the Orbitide **2** and **1** coding regions.

At the amino acid level, the putative linear transcript in ‘Z 11637’ consists of a fusion of the first three amino acid residues of **2** (MLI), the conserved residues PPFF, contributed either entirely from **1** or **2** or as a portion from both, and the last two residues of **1** (LI) (Fig. 5). The amplified fragment in ‘Hollandia’ is similar to that from ‘Z 11637’ and, as a result, the alignment of ‘CDC Bethune’ with ‘Hollandia’ is identical to that with ‘Z11637’. There were no mutations observed in the DNA coding fragment of **8** which explains the comparable expression of **8** among ‘Hollandia’, ‘Z 11637’ and ‘CDC Bethune’. The putative precursor of **1**, **7** and **18** is flanked by “DG” and “FGK” amino acid residues at the amino and carboxy termini, respectively. New orbitide **18** is the result of the removal of a whole repeat unit suggesting that orbitide expression is conserved and that a mechansim(s) to delete fragments function(s) to maintain the integrity of the orbitide repeat units. A search for similar, whole orbitide unit, insertions/deletions in the other main orbitide gene (*LINUSORB A*) to determine whether a consistent mechanism for orbitide gene recombination exists is underway.

The mutation event occurring in ‘Hollandia’ and ‘Z 11637’ was determined to be heritable by crossing ‘Hollandia’ with ‘CDC Sorrel’ and ‘Hollandia’ with ‘CDC Sanctuary’. The sequence of *LINUSORB B* in ‘CDC Sorrel’ and ‘CDC Sanctuary’ is identical to that of ‘CDC Bethune’, and, as such, these cultivars expressed **1**, **2**, **5**, **7**, **10** and **14** at similar concentrations to the check ‘CDC Bethune’. Inheritance of the altered *LINUSORB B* gene was observed both phenotypically (via HPLC-DAD and mass spectral analyses) and genotypically (PCR and qPCR) in F_3_ seed. The expected Mendelian ratios for monogenetic inheritance were observed in this population. No distortion of the expected phenotypic ratios was observed in reciprocal crosses in these populations, indicating that maternal factors do not affect orbitide expression.

Although it is difficult to determine the timeline of the DNA variation, we infer that the deletion event probably occurred in a common ancestor of ‘Hollandia’ and ‘Z 11637’ as both orignated from the Netherlands. Rapid changes in the flax genome as a response to growth environment has been reported in which DNA variants can be stable or unstably inherited [29]. ‘Hollandia’ is a flax cultivar in which stable genomic variations have been observed, others including ‘Stormont Cirrus’, ‘Rembrandt’, and ‘Liral Monarch’ [30]. CA Cullis [30] has suggested that organization of the flax genome contributes to widespread genomic variations that may be observed in flax within a generation. We speculate that the deletion event described here could be an example of this type of genomic reorganization induced by environmental stress. It is also intriguing to speculate on the role of orbitides in flax physiology and agronomic selection. All flax cultivars and accessions examined in the FCC expressed orbitides in their seed [23], however, as demonstrated in this paper, significant alterations in their composition and total concentration are possible.

## Conclusions

The unidentified peak observed in HPLC-DAD chromatograms of extracts from ‘Hollandia’ and ‘Z 11637’ flax cultivars is attributed to novel orbitide **18**. Mass spectrometry (high resolution HPLC-ESI-MS and HPLC-ESI-MS/MS) and NMR spectroscopy revealed the structure of **18** as [1−9-N*α*C]-*O*LIPPFFLI with *m/z* 1072.6. The analysis of the gene sequence of *LINUSORB B* in ‘Hollandia’ and ‘Z 11637’ explains the apparently reduced total orbitide concentration by the lack of **1** and **2**, and the expression of **18**, as compared with ‘CDC Bethune’. Sequencing results also corroborate the amino acid sequence of **18** deduced from spectroscopy. Future work will include recovery of sequences with similarity to *LINUSORB B* and *LINUSORB A* from the entire FCC [24] which covers approximately 95% of the genetic variation in flax. Correlations between gene structure and orbitide expression will be made once this data is obtained.

## Methods

### Flaxseed

Seed from the cultivars ‘Hollandia’ (CN 98056), ‘Z 11637’ (CN 98150), and ‘CDC Bethune’ were regenerated at Kernen Crop Research Farm, Saskatoon, SK and Morden Research Station, Morden, MB in 2009, 2010, and 2011. Flaxseed was kindly donated by Dr. Scott Duguid (Morden Research Station, MB) and Drs. Helen Booker and Gordon Rowland (Crop Development Centre, Saskatoon SK).

### Sample preparation for orbitide composition analysis

Approximately 120 mg of flaxseed from each accession for each growing season was weighed and placed directly into wells (~ 1.8 mL volume) of 96-well plates. Each sample was prepared according to Burnett et al. [23] in quadruplicate to enable replicate composition analyses for oxidized and unoxidized (native) extracts. Specifically, pre-heated H_2_O (60 °C) was added to each well at a seed˗water ratio of 1:10 (*w*/*v*) and wells were covered with a 96-well plate sealing mat (square cap). Covered plates were incubated at 60 °C for 2 h and then gum extracts were removed *via* pipette. The degummed seeds were ground at 1400 strokes per min for 10 min using a 2010 Geno/grinder (SPEX CertiPrep, Inc., Methucen, NJ) and spherical 5-mm zirconia grinding media (1 per well). A 100 μL aliquot of internal standard [1−9-N*α*C],[1-Abu]-linusorb B2 **(**0.1 mg/mL in MeOH**)**, prepared as previously reported, [31] was added to each well. Subsequently, 860 μL of methanol:water (78:22, v/v) was added to each well to make a final dilution of 1:8 (w/v). Samples were mixed for 2 min at 1400 strokes per min and were subsequently incubated at 60 °C for 2 h. Samples were centrifuged at 1760×g for 20 min and a 400 μL aliquote of the resulting supernatant was transferred to a 96-well filter plate.

Filter plates were covered and placed on top of receiving 96-well plates and assembled plates were placed in a centrifuge for filtration at 1760×g for 10 min. Filtered extracts (100 μL aliquots) were transferred to 96-well HPLC trays and were subsequently oxidized with H_2_O_2_ (50 μL, 4.5% in H_2_O, v/v) or diluted with H_2_O (50 μL) such that each cultivar per harvest year had replicates for each treatment. After 1 h at room temperature, the oxidized extracts were quenched with Na_2_S_2_O_3_ (150 μL, 0.2 M) in 70% aq. MeOH, whereas 70% aq. MeOH (150 μL) was added to the diluted extracts. The 96-well trays were placed in an HPLC-DAD, as described below, with an autoinjector for sample analyses. Extracts were also analyzed *via* high resolution HPLC Electrospray Ionization mass spectrometry (HR-HPLC-ESI-MS and HPLC-ESI–MS/MS).

### Extraction and isolation

Orbitides were extracted from ‘Hollandia’ (CN 98056) and ‘Z 11637’ (CN 98150) seed regenerated in 2009. Seed samples from Saskatoon, SK and Morden, MB, were combined based on flax accession (total mass of extracted flaxseed was 80 g) and were ground prior to extraction with 70% MeOH (1:20 v/v, × 2). Each sample was incubated at 60 °C for 2 h. Aliquots of each aqueous methanolic extract were filtered through 0.45 μm PTFE membranes for HPLC-DAD and HPLC-ESI-MS analyses. Then, extracts were freed of solvent using a rotary evaporator to yield crude orbitide extracts (total mass 4.95 g). Mass spectra of all ‘Hollandia’ and ‘Z 11637’ extracts indicated the presence of **18** possessing quasimolecular ion peak at *m/z* 1072.6, in addition to other known orbitides.

Conversion of methionine-containing orbitides to their methionine *S*-oxide-containing form is necessary to simplify orbitide peak chromatographic separation, and increase the concentration of novel orbitides in a single oxidation state. Consequently, crude orbitide extracts were oxidized with H_2_O_2_ prior to flash column chromatography as follows. Extracts were suspended in MeOH (5 mL, × 2), filtered through 0.45 *μ*m PTFE membranes, and subsequently combined with H_2_O_2_ (1 mL, 30% in H_2_O, v/v). Oxidation was monitored by HPLC-DAD and upon conversion of **18** to **19**, the reaction mixture was quenched with saturated Na_2_S_2_O_3_ in 70% aq. MeOH (10 mL), then filtered. The sample was freed of solvent using a rotary evaporator (dry mass of sample 4.56 g). The oxidized extract was subjected to flash column chromatography on silica gel 60 (40-63 μm particle size, EMD Chemicals). Sequential elution with the following solvent systems was conducted (each 200 mL, with each eluent being divided into two fractions): (a) *n*-hexane; (b) 20% EtOAc in *n*-hexane; (c) 50% EtOAc in *n*-hexane; (d) 80% EtOAc in *n*-hexane; (e) 100% EtOAc; (f-j) 2% MeOH in CH_2_Cl_2_ to 10% MeOH in CH_2_Cl_2_ using 2% MeOH increments. Each fraction collected was analyzed via HPLC-DAD and HPLC-ESI-MS. Fractions determined to contain substantial amounts of **19** (*m/z* 1088.6) were purified using preparative reversed-phase chromatography.

### HPLC-DAD analyses

#### Analytical

Analyses of FCC orbitide composition were performed on a 1200 series HPLC system (Agilent Technologies Canada, Mississauga, ON) equipped with a quaternary pump, autosampler, photodiode-array detector (wavelength range 190-300 nm), and a degasser. Chromatographic separations were carried out on 50 mm × 4.6 mm i.d., reversed phase Chromolith SpeedRod RP-18e columns (Merck KGaA, Darmstadt, Germany) equipped with in-line filters. The mobile phase consisted of a linear gradient of H_2_O and acetonitrile (CH_3_CN) (70:30 to 30:70 in 4 min, to 10:90 in 0.5 min, to 70:30 in 0.5 min, to equilibration for 1 min) at a flow rate of 2 mL/min [31]. All analyses were conducted at 23 °C using 10 μL injection volumes and recording absorbance over the entire UV spectrum. Peak area integration was conducted at a wavelength of 214 nm with a 10 nm bandwidth. All samples analyzed via HPLC-DAD and HPLC-MS were previously filtered through 0.45 μm PTFE membranes. The molar extinction coefficients at 214 nm of all orbitides discussed in this manuscript are similar and, therefore, A_214_ is proportional to orbitide concentration. Pure orbitide standards were not used to determine either matrix effects or concentration. All concentrations are compared based on their A_214_ during elution from an HPLC column.

#### Preparative

Preparative reversed phase chromatography was performed on an Agilent 1200 series HPLC system (Agilent Technologies Canada, Mississauga, ON) equipped with a Chromolith® SemiPrep RP-18e column (100 × 10 mm i.d.), and a U*V*/VIS detector operating at a wavelength of 214 nm. The mobile phase consisted of H_2_O–CH_3_CN (55:45 for 12 min, to 5:95 in 1.0 min, to 55:45 in 1.0 min, to equilibration for 6 min) at a flow rate of 3 mL/min. All preparative separations were performed at ambient temperature.

### Mass spectral analyses

High resolution HPLC-ESI-MS and HPLC-ESI–MS/MS analyses were performed on an Agilent 1200 series HPLC system connected directly to a micrOTOF-Q II hybrid quadrupole time of flight MS/MS (Bruker Daltonik GmbH, Bremen, Germany) with Apollo II electrospray ionization (ESI) ion source at a capillary voltage of -4500 V, nebulizer gas at 4.0 bar, and drying gas temperature held at 200 °C. Chromatographic separation for MS analyses was achieved at ambient temperature using a Chromolith^®^ FastGradient RP-18e column (50 mm × 2.0 mm i.d., Merck KGaA, Darmstadt, Germany). The mobile phase consisted of a linear gradient of 0.1% formic acid in H_2_O and 0.1% formic acid in CH_3_CN (60:40 for 2 min, to 10:90 in 8 min, to 60:40 in 0.5 min, to equilibration for 5.5 min) at a flow rate of 0.4 mL/min [16]. HPLC-ESI-MS/MS analyses were conducted on a Bruker micrOTOF-Q II Mass Spectrometer using identical parameters to those described for HR-HPLC-ESI-MS.

### Structural analysis via nuclear magnetic resonance (NMR)

Proton NMR spectra were recorded on a 600 MHz Bruker Avance spectrometer (5 mm PABBO BB-probe head; TopSpin 3.2 software). The ^1^H NMR spectra (600 MHz) chemical shifts (δ) values are reported in parts per million (ppm) relative to the internal standard TMS. The δ values are referenced to CDCl_3_ at 7.26 ppm, and multiplicities are indicated by the following symbols: s = singlet, d = doublet, dd = doublet of doublets, m = multiplet, and br = broad. For ^13^C NMR (125.8 MHz), the chemical shift (δ) values were referenced to CDCl_3_ (77.23 ppm).

### Orbitide gene sequence analyses

Primers were designed to amplify the portions of *LINUSORB B* which encoded for Orbitides **1**, **3**, and **8**. The sequence of this gene has been confirmed in previous work [1–3], as well as other sources, and the identifier, *LINUSORB B*, is used throughout this paper to maintain consistency with our proposed systematic nomenclature [22]. Flax accessions and cultivars examined for sequence analysis were ‘Hollandia’, ‘Z 11637’, and ‘CDC Bethune’. A quick alkaline lysis method was used to extract crude total DNA from leaves of 14 day old seedlings [32]. Crude extracts (1 μL) were used as templates for PCR amplification of a 369 bp fragment using forward (5’ATTTCTGGAAAGGATGGCGG3’) and reverse (5’CTTGTCACCCTGCTGCTC3’) primers with annealing temperatures of 58.2 and 58.0 °C, respectively. The sizes of the fragments from different accessions were compared using agarose gel electrophoresis. The fragments from two PCRs (each 25 μL) were purified using a Qiagen DNAesy PCR purification kit and were then sequenced using the amplification primers or one of two internal primers (5’CGGCATTATTATACTTGTGGCCG3’ or 5’CGGCCACAAGTATAATAATGCCG3’). PCR fragment sequencing was performed by the National Research Council (NRC) Canada, Saskatoon, DNA Sequencing Facility. PCR product sequences were aligned against the *LINUSORB B* sequence from ‘CDC Bethune’ using Geneious 6.0 (Biomatters Inc., Auckland, New Zealand).

## Additional files

Additional file 1: **Table S1.** Calculated flaxseed orbitide masses and sequences. (DOCX 22 kb)

Additional file 2: **Figure S1.** ^1^H NMR spectrum of [1−9-N*α*C]-*O*LIPPFFLI (**19**). (PDF 178 kb)

Additional file 3: **Figure S2.** ^13^C NMR spectrum of [1−9-N*α*C]-*O*LIPPFFLI (**19**). (PDF 145 kb)

Additional file 4: **Figure S3.** ^1^H-^1^H COSY spectrum of [1−9-N*α*C]-*O*LIPPFFLI (**19**). (PDF 479 kb)

Additional file 5: **Figure S4.** DEPT spectrum of [1−9-N*α*C]-*O*LIPPFFLI (**19**). (PDF 145 kb)

Additional file 6: **Figure S5.** ^1^H-^13^C HMBC spectrum of [1−9-N*α*C]-*O*LIPPFFLI (**19**). (PDF 189 kb)

Additional file 7: **Figure S6.** ^1^H-^13^C HSQC spectrum of [1−9-N*α*C]-*O*LIPPFFLI (**19**). (PDF 229 kb)

Additional file 8: **Figure S7.** ^1^H-^1^H NOESY spectrum of [1−9-N*α*C]-*O*LIPPFFLI (**19**). (PDF 476 kb)

Additional file 9: **Figure S8.** ^1^H-^1^H TOCSY spectrum of [1−9-N*α*C]-*O*LIPPFFLI (**19**). (PDF 763 kb)

Additional file 10: Hollandia,Bethune_Orbitide_seq.fa sequence obtained from Sanger sequencing of the Hollandia/Z11637 orbitide [**18**, **8**] PCR fragment and the CDC Bethune orbitide [**1**, **3**, **8**] PCR fragment. (FA 717 bytes)

## Abbreviations

CDC: Crop Development Centre
CN: Canadian National
COSY: Correlation spectroscopy
FCC: Flax core collection
HMBC: Heteronuclear multiple-bond correlation
HMQC: Heteronuclear multiple quantum correlation
HPLC-DAD: High performance liquid chromatography with diode array detector
HPLC-ESI-MS/MS: High resolution high performance liquid chromatography with electrospray ionization tandem mass spectrometry
HR-HPLC-ESI-MS: High resolution high performance liquid chromatography with electrospray ionization mass spectrometry
NCBI: National Center for Biotechnology Information
NMR: Nuclear Magnetic Resonance
NOESY: Nuclear Overhauser effect spectroscopy
PGRC: Plant Gene Resources of Canada
TOCSY: Total correlation spectroscopy
