# Supplementary material for: Novel flax orbitide derived from genetic deletion
Source: BMC Plant Biol. 2018 May 21;18:90. doi: 10.1186/s12870-018-1303-8 (PMC5963108; doi:10.1186/s12870-018-1303-8)
Supplement: Supplementary file 1 — Table S1. Calculated flaxseed orbitide masses and sequences. (DOCX 22 kb) [file 12870_2018_1303_MOESM1_ESM.docx]

**Table S1** Calculated flaxseed orbitide masses and sequences

| Structure No. | Orbitide name*^a^* | Molecular formula | Mass ([M + H]^+^, Da) | | Amino acid sequence*^b^* |
| --- | --- | --- | --- | --- | --- |
|  |  |  | Exp. | Calc. |  |
| **1** | [1−9-N*α*C]-linusorb B3 | C_57_H_86_N_9_O_9_ | 1040.6672*^c^* | 1040.6543 | [1−9-N*α*C]-ILVPPFFLI |
| **2** | [1−9-N*α*C]-linusorb B2 | C_56_H_84_N_9_O_9_S | 1058.6236*^c^* | 1058.6107 | [1−9-N*α*C]-MLIPPFFVI |
| **3** | [1−9-N*α*C],[1-MetO*^d^*]-linusorb B2 | C_56_H_84_N_9_O_10_S | 1074.6201*^c^* | 1074.6056 | [1−9-N*α*C]-*O^e^*LIPPFFVI |
| **4** | [1−9-N*α*C],[1-MetO_2_*^f^*]-linusorb B2 | C_56_H_84_N_9_O_11_S | 1090.5943*^g^* | 1090.6006 | [1−9-N*α*C]-J*^h^*LIPPFFVI |
| **5** | [1−8-N*α*C]-linusorb A2 | C_57_H_78_N_9_O_8_S | 1048.5838*^c^* | 1048.5689 | [1−8-N*α*C]-MLLPFFWI |
| **6** | [1−8-N*α*C],[1-MetO]-linusorb A2 | C_57_H_78_N_9_O_9_S | 1064.5681*^c^* | 1064.5638 | [1−8-N*α*C]-*O*LLPFFWI |
| **7** | [1−8-N*α*C]-linusorb B1 | C_51_H_77_N_8_O_8_S | 961.5705*^c^* | 961.5580 | [1−8-N*α*C]-MLVFPLFI |
| **8** | [1−8-N*α*C],[1-MetO]-linusorb B1 | C_51_H_77_N_8_O_9_S | 977.5665*^c^* | 977.5529 | [1−8-N*α*C]-*O*LVFPLFI |
| **9** | [1−8-N*α*C],[1-MetO_2_]-linusorb B1 | C_51_H_77_N_8_O_10_S | 993.5303*^g^* | 993.5478 | [1−8-N*α*C]-JLVFPLFI |
| **10** | [1−8-N*α*C]-linusorb A3 | C_55_H_74_N_9_O_8_S_2_ | 1052.5191*^c^* | 1052.5096 | [1−8-N*α*C]-MLMPFFWV |
| **11** | [1−8-N*α*C],[3-MetO]-linusorb A3 | C_55_H_74_N_9_O_9_S_2_ | 1068.5185*^c^* | 1068.5045 | [1−8-N*α*C]-ML*O*PFFWV |
| **12** | [1−8-N*α*C],[1-MetO]-linusorb A3 | C_55_H_74_N_9_O_9_S_2_ | 1068.5022*^i^* | 1068.5045 | [1−8-N*α*C]-*O*LMPFFWV |
| **13** | [1−8-N*α*C],[1,3-MetO]-linusorb A3 | C_55_H_74_N_9_O_10_S_2_ | 1084.4951*^i^* | 1084.4995 | [1−8-N*α*C]-*O*L*O*PFFWV |
| **14** | [1−8-N*α*C]-linusorb A1 | C_56_H_76_N_9_O_8_S_2_ | 1066.5387*^c^* | 1066.5253 | [1−8-N*α*C]-MLMPFFWI |
| **15** | [1−8-N*α*C],[1-MetO]-linusorb A1 | C_56_H_76_N_9_O_9_S_2_ | 1082.5329*^c^* | 1082.5202 | [1−8-N*α*C]-*O*LMPFFWI |
| **16** | [1−8-N*α*C],[3-MetO]-linusorb A1 | C_56_H_76_N_9_O_9_S_2_ | 1082.5330*^c^* | 1082.5202 | [1−8-N*α*C]-ML*O*PFFWI |
| **17** | [1−8-N*α*C],[1,3-MetO]-linusorb A1 | C_56_H_76_N_9_O_10_S_2_ | 1098.5299*^c^* | 1098.5151 | [1−8-N*α*C]-*O*L*O*PFFWI |
| **18** | [1−9-N*α*C]-linusorb F1 | C_57_H_86_N_9_O_9_S | 1072.6246 | 1072.6264 | [1−9-N*α*C]-MLIPPFFLI |
| **19** | [1−9-N*α*C],[1-MetO]-linusorb F1 | C_57_H_86_N_9_O_10_S | 1088.6211 | 1088.6213 | [1−9-N*α*C]-*O*LIPPFFLI |
| **20** | [1−9-N*α*C],[1-MetO_2_]-linusorb F1 | C_57_H_86_N_9_O_11_S | 1104.6150 | 1104.6162 | [1−9-N*α*C]-JLIPPFFLI |
| **21** | [1−9-N*α*C]-linusorb C1 | C_66_H_88_N_11_O_9_S | 1210.6638*^c^* | 1210.6482 | [1−9-N*α*C]-MLKPFFFWI |
| **22** | [1−9-N*α*C],[1-MetO]-linusorb C1 | C_66_H_88_N_11_O_10_S | 1226.6581*^c^* | 1226.6431 | [1−9-N*α*C]-*O*LKPFFFWI |
| **23** | [1−9-N*α*C]-linusorb D1 | C_54_H_77_N_10_O_10_ | 1025.5946*^c^* | 1025.5819 | [1−9-N*α*C]-GIPPFWLTL |
| **24** | [1−10-N*α*C]-linusorb E3 | C_59_H_89_N_10_O_10_ | 1097.6746*^j^* | 1097.6758 | [1−10- N*α*C]-GILVPPFFLI |
| **25** | [1−10-N*α*C]-linusorb E2 | C_58_H_87_N_10_O_10_S | 1115.6307 *^j^* | 1115.6322 | [1−10- N*α*C]-GMLIPPFFVI |
| **26** | [1−10-N*α*C],[2-MetO]-linusorb E2 | C_58_H_87_N_10_O_11_S | 1131.6229 *^j^* | 1131.6271 | [1−10- N*α*C]-G*O*LIPPFFVI |
| **27** | [1−9-N*α*C]-linusorb E1 | C_53_H_80_N_9_O_9_S | 1018.5873 *^j^* | 1018.5794 | [1−9- N*α*C]-GMLVFPLFI |
| **28** | [1−9-N*α*C],[2-MetO]-linusorb E1 | C_53_H_80_N_9_O_10_S | 1034.5787 *^j^* | 1034.5743 | [1−9- N*α*C]-GOLVFPLFI |

*^a^* New systematic nomenclature proposed by Shim *et al*. (2015).

*^b^* [1−#-N*α*C] describes N to C linkage through the *α*-amino group between amino acid 1 and amino acid ‘#’.

*^c^* Experimental masses reported by Owiti *et al*. (2014).

*^d^* Designation MetO describes methionine *S*-oxide.

*^e^* Amino acid symbol *O* used for methionine *S*-oxide.

*^f^* Designation MetO_2_ describes methionine *S*,*S*-dioxide.

*^g^* Experimental masses reported by Jadhav *et al*. (2013).

*^h^* Amino acid symbol J used for methionine *S*,*S*-dioxide.

*^i^* Experimental masses reported by Burnett *et al*. (2016).

*^j^* Experimental masses reported by Burnett *et al*. (2015).
