# Supplementary figures and images for: Novel flax orbitide derived from genetic deletion
Source: BMC Plant Biol. 2018 May 21;18:90. doi: 10.1186/s12870-018-1303-8 (PMC5963108; doi:10.1186/s12870-018-1303-8)

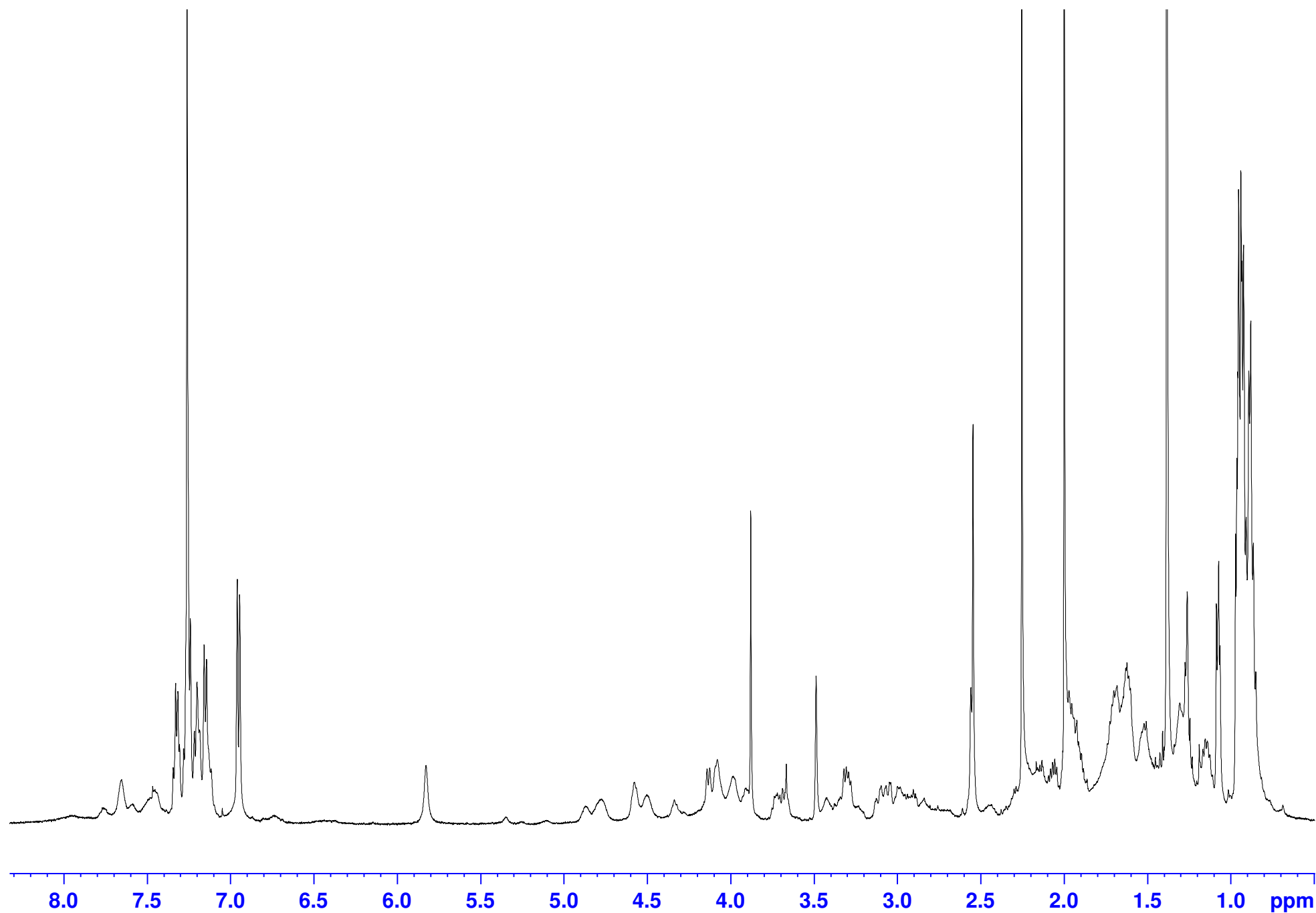

**Figure S1.**  $^1\text{H}$  NMR spectrum of [1-9-NaC]-OLIPFFLI (19).

Supplement: Supplementary file 2 — Figure S1. 1H NMR spectrum of [1−9-NαC]-OLIPPFFLI (19). (PDF 178 kb) [file 12870_2018_1303_MOESM2_ESM.pdf]

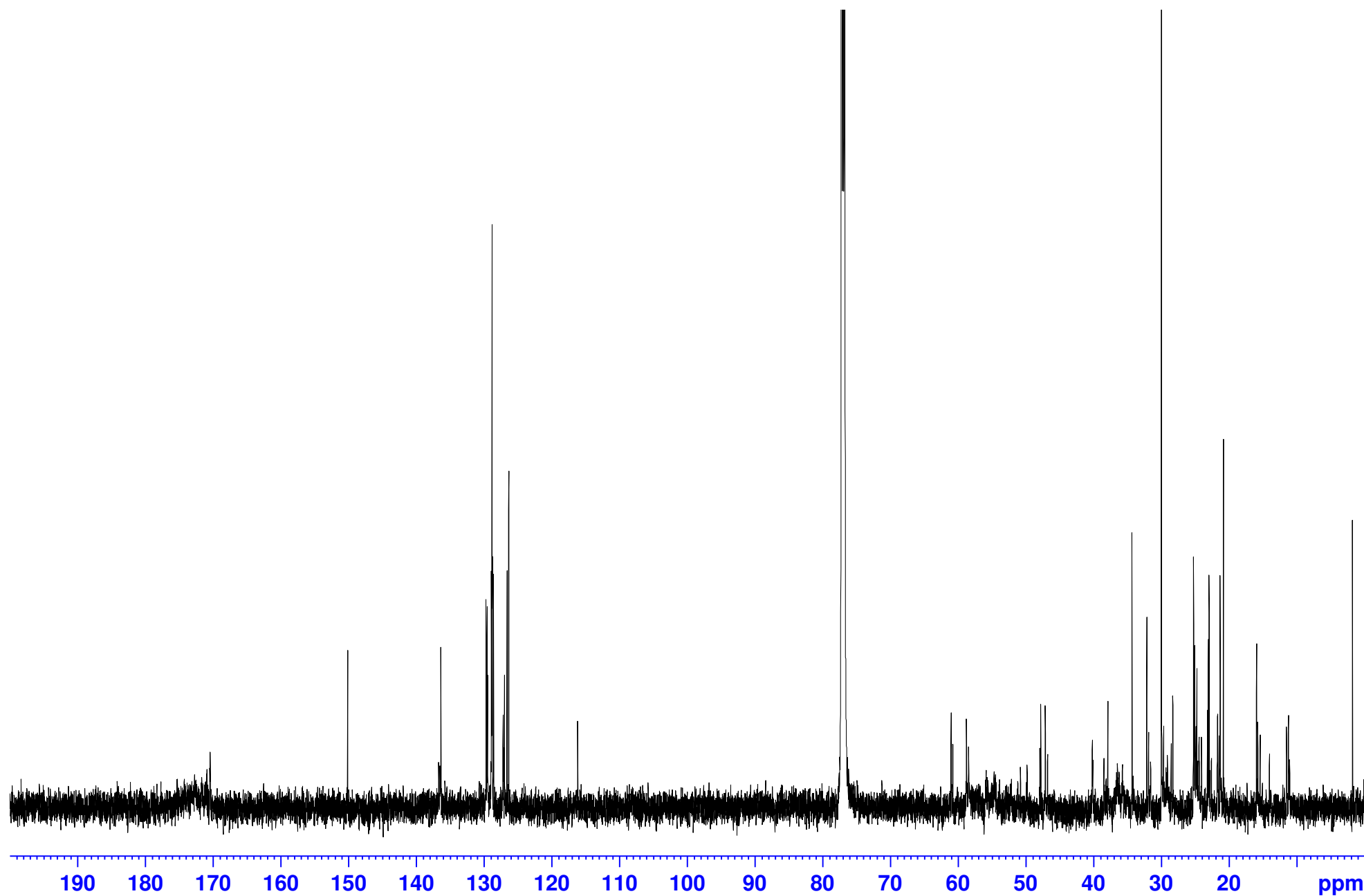

**Figure S2.**  $^{13}\text{C}$  NMR spectrum of [1-9-N $\alpha$ C]-OLIPPFFLI (19).

Supplement: Supplementary file 3 — Figure S2. 13C NMR spectrum of [1−9-NαC]-OLIPPFFLI (19). (PDF 145 kb) [file 12870_2018_1303_MOESM3_ESM.pdf]

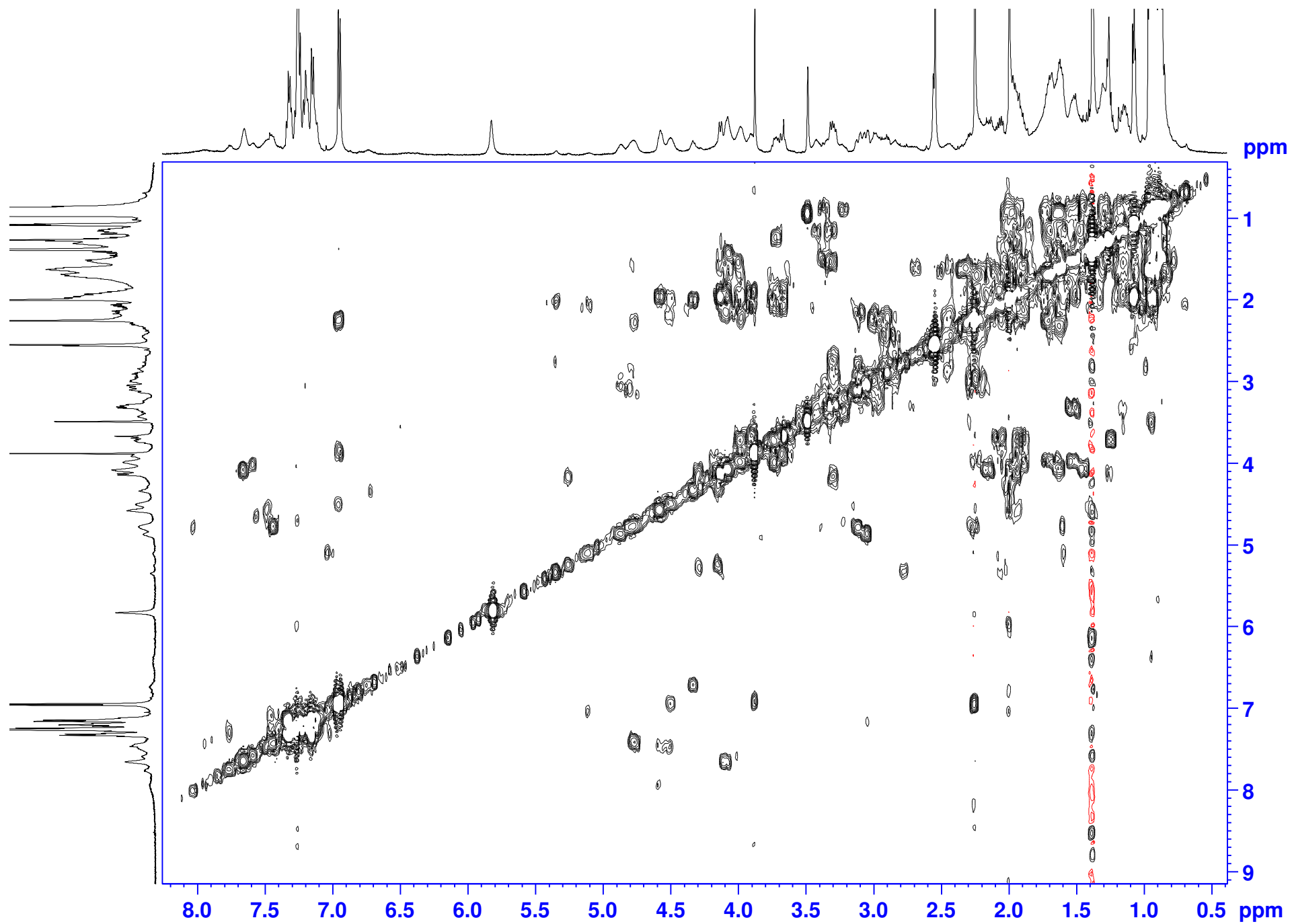

**Figure S3.**  $^1\text{H}$ - $^1\text{H}$  COSY spectrum of [1-9-NaC]-OLIPPFLLI (19).

Supplement: Supplementary file 4 — Figure S3. 1H-1H COSY spectrum of [1−9-NαC]-OLIPPFFLI (19). (PDF 479 kb) [file 12870_2018_1303_MOESM4_ESM.pdf]

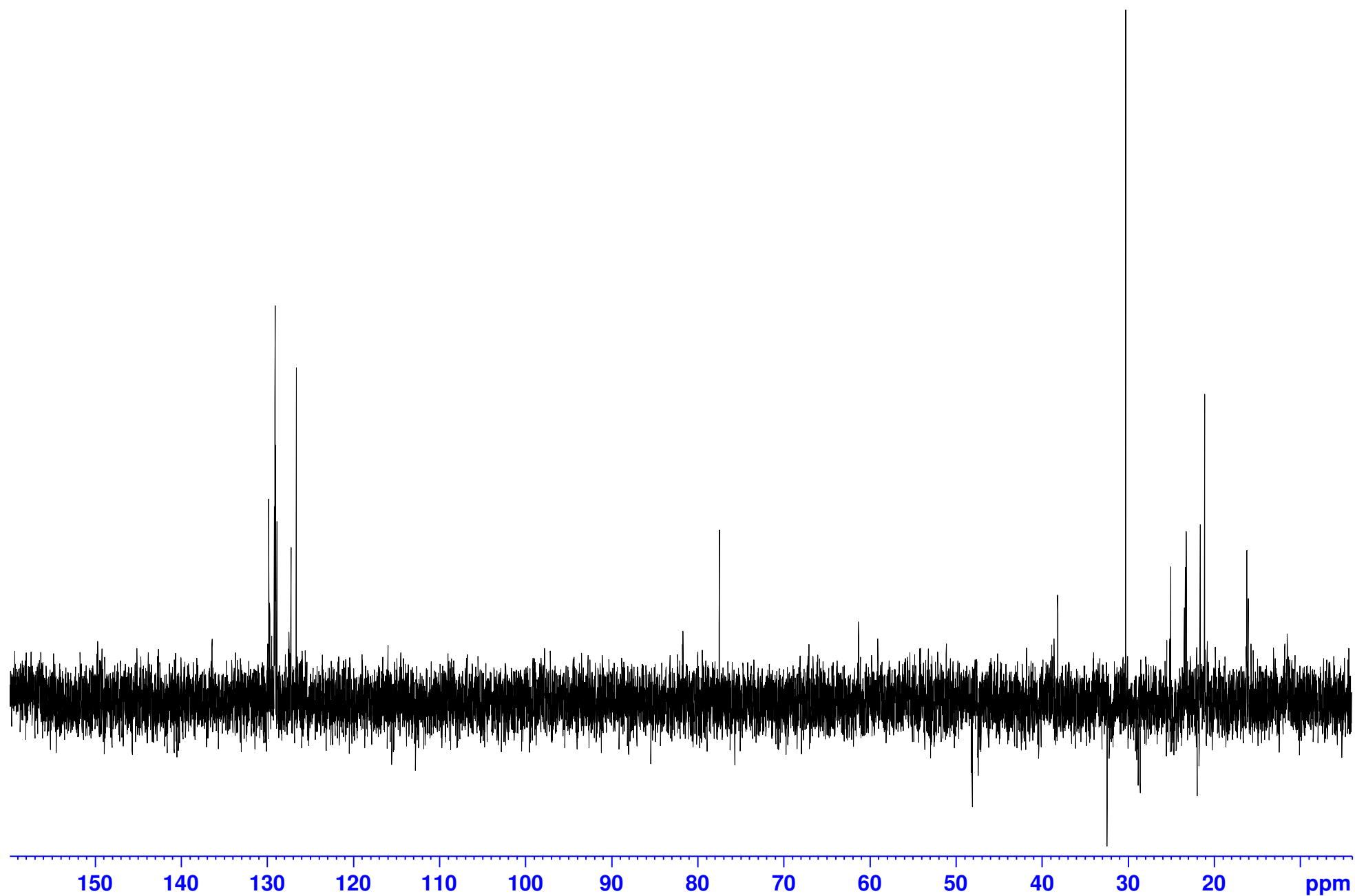

**Figure S4.** DEPT spectrum of [1-9-N $\alpha$ C]-OLIPPFLLI (19).

Supplement: Supplementary file 5 — Figure S4. DEPT spectrum of [1−9-NαC]-OLIPPFFLI (19). (PDF 145 kb) [file 12870_2018_1303_MOESM5_ESM.pdf]

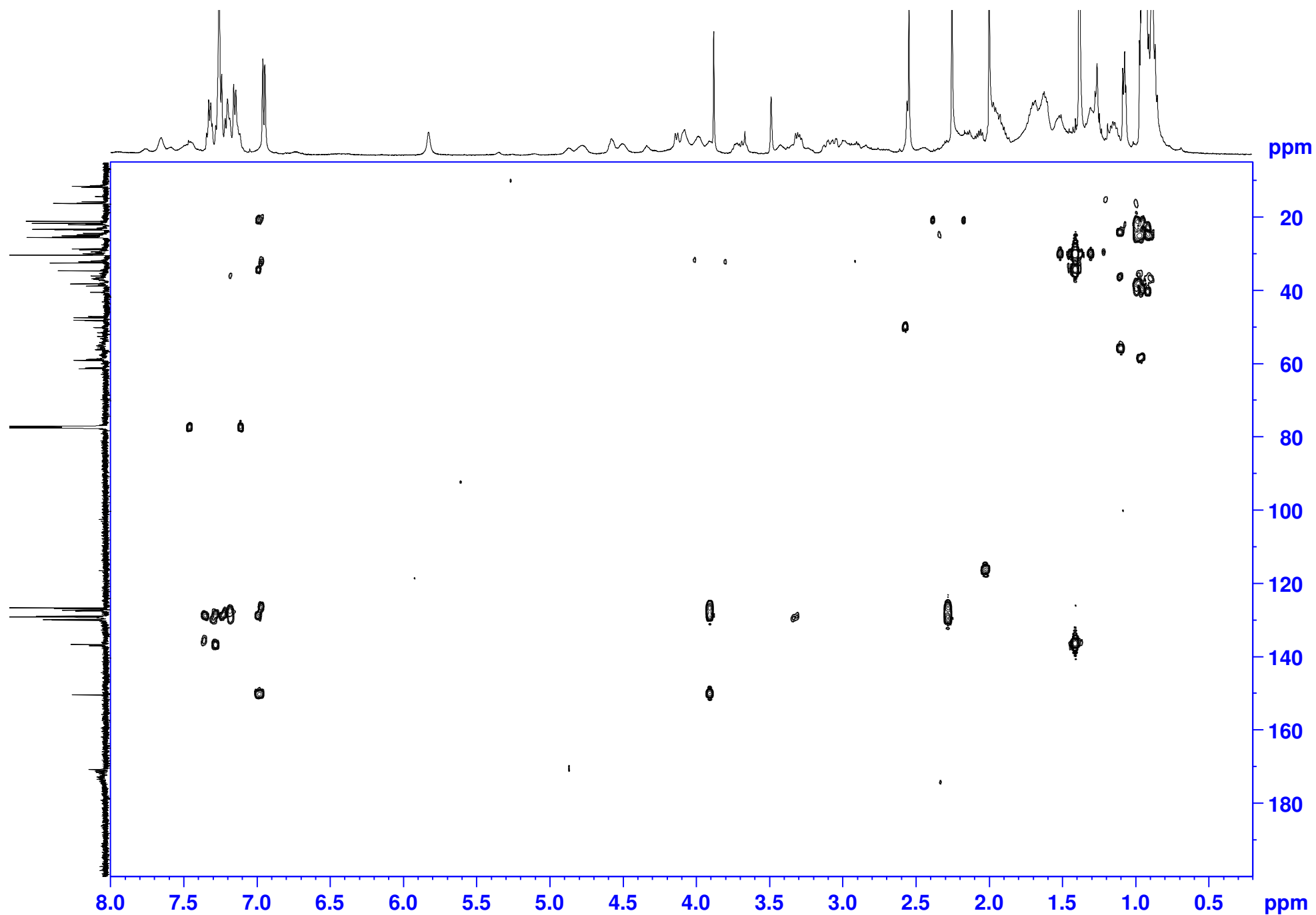

Figure S5.  $^1\text{H}$ - $^{13}\text{C}$  HMBC spectrum of [1-9-NaC]-OLIPFFLI (19).

Supplement: Supplementary file 6 — Figure S5. 1H-13C HMBC spectrum of [1−9-NαC]-OLIPPFFLI (19). (PDF 189 kb) [file 12870_2018_1303_MOESM6_ESM.pdf]

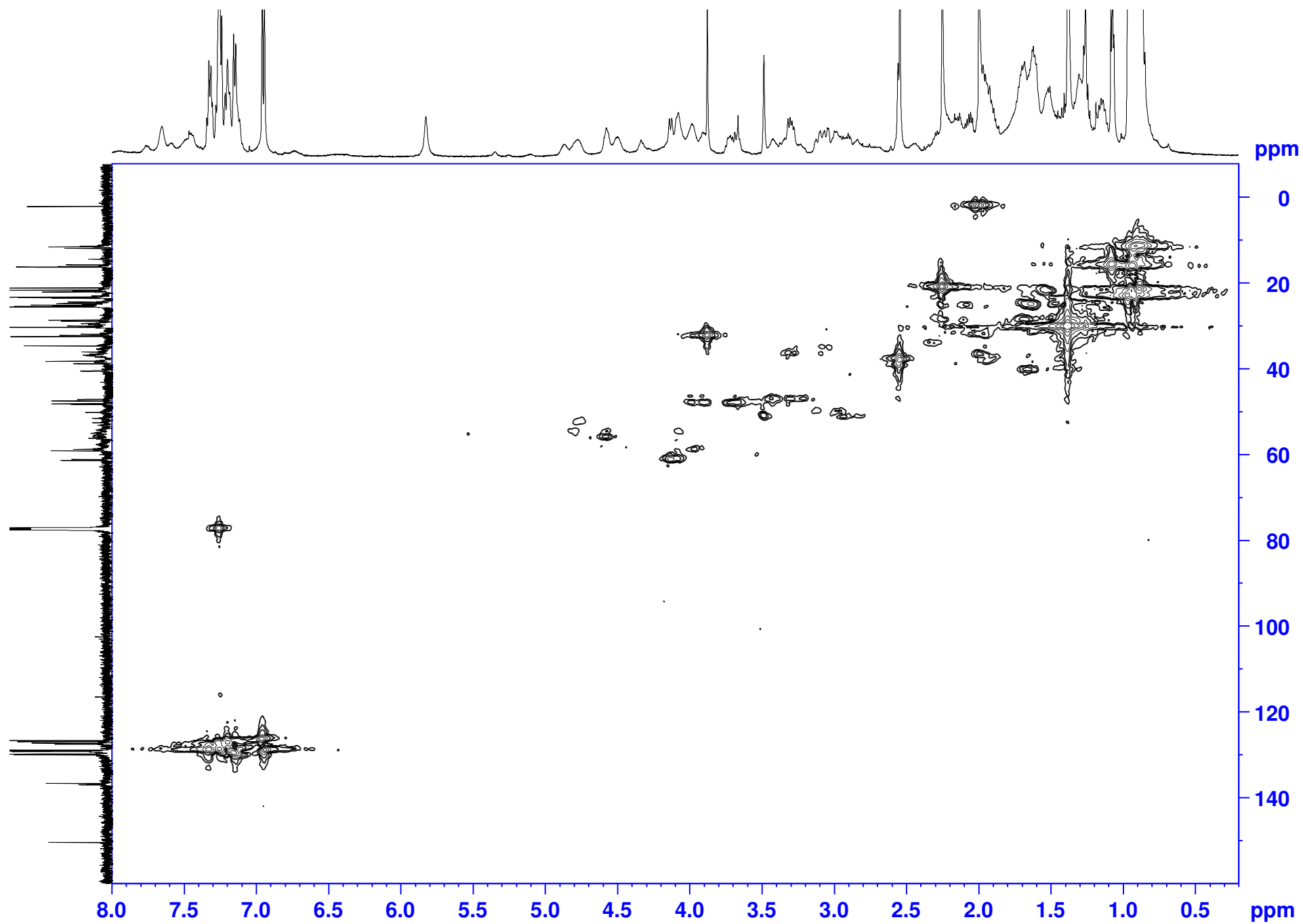

**Figure S6.**  $^1\text{H}$ - $^{13}\text{C}$  HSQC spectrum of [1-9-NaC]-OLIPPFFLI (19).

Supplement: Supplementary file 7 — Figure S6. 1H-13C HSQC spectrum of [1−9-NαC]-OLIPPFFLI (19). (PDF 229 kb) [file 12870_2018_1303_MOESM7_ESM.pdf]

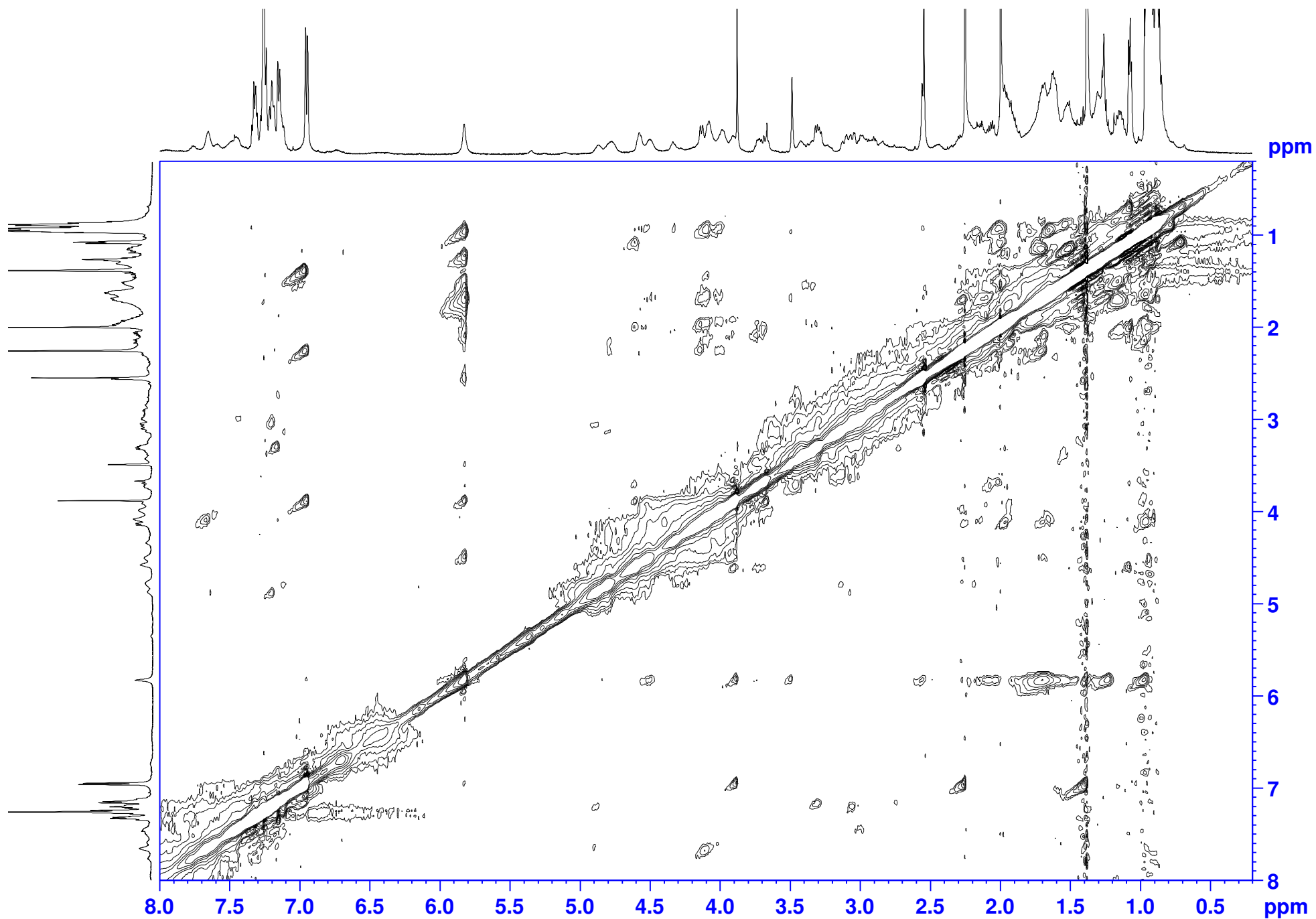

**Figure S7.**  $^1\text{H}$ - $^1\text{H}$  NOESY spectrum of [1-9-NaC]-OLIPFFLI (19).

Supplement: Supplementary file 8 — Figure S7. 1H-1H NOESY spectrum of [1−9-NαC]-OLIPPFFLI (19). (PDF 476 kb) [file 12870_2018_1303_MOESM8_ESM.pdf]

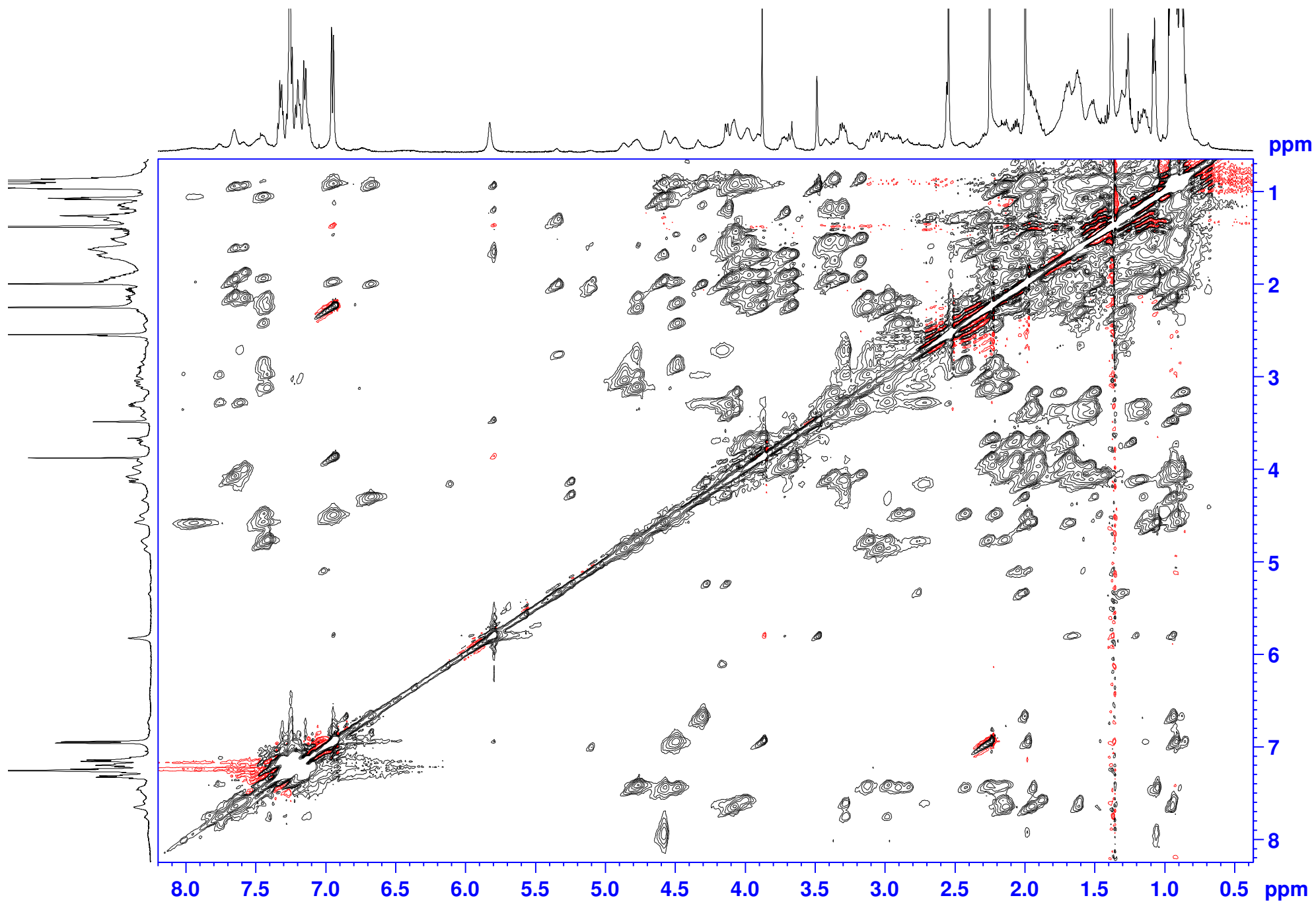

Figure S8.  $^1\text{H}$ - $^1\text{H}$  TOCSY spectrum of [1-9-NaC]-OLIPPFLLI (19).

Supplement: Supplementary file 9 — Figure S8. 1H-1H TOCSY spectrum of [1−9-NαC]-OLIPPFFLI (19). (PDF 763 kb) [file 12870_2018_1303_MOESM9_ESM.pdf]
